# Evaluating the preoperative diagnostic value of ^99m^Tc‐MIBI/^99m^TcO_4_^−^ dual‐isotope SPECT/CT fusion imaging and ultrasonography in suspected primary hyperparathyroidism

**DOI:** 10.1002/acm2.70763

**Published:** 2026-08-27

**Authors:** Yanmei Li, Danna Hou, Zhehao Lyu, Xingrong Lan, Ziyue Zhang, Yu Lu, Peng Fu, Huiqi Gao

**Affiliations:** ^1^ Department of Nuclear Medicine Harbin Medical University Harbin China

**Keywords:** normocalcemic primary hyperparathyroidism, PECT/CT, preoperative localization, primary hyperparathyroidism

## Abstract

**Background:**

Primary hyperparathyroidism (PHPT) is a common endocrine disorder that often requires surgical intervention. Preoperative localization of parathyroid lesions is critical for successful surgery. This study compares the diagnostic value of ^99m^Tc‐MIBI/^99m^TcO_4_^−^ dual‐isotope SPECT/CT fusion imaging and ultrasonography (US) in patients with suspected PHPT, and explores the correlation between lesion volume and preoperative serum biochemical markers.

**Objective:**

This study aimed to assess the preoperative diagnostic performance of US and ^99m^Tc‐MIBI/^99m^TcO_4_^−^ dual‐isotope SPECT/CT fusion imaging in PHPT and investigate the relationship between serum parameters and parathyroid adenoma volume.

**Methods:**

A retrospective analysis was conducted on 75 patients with elevated parathyroid hormone (PTH) levels and suspected primary hyperparathyroidism between September 2023 and November 2024. All patients underwent ^99m^Tc‐MIBI/^99m^TcO_4_^−^ dual‐isotope SPECT/CT fusion imaging and US. Using surgical histopathology as the gold standard, the sensitivity and positive predictive value (PPV) of both imaging modalities in detecting parathyroid lesions were calculated. Additionally, a preliminary analysis was performed to assess the relationship between lesion volume and preoperative serum biochemical markers.

**Results:**

Among the 75 patients, 68 were confirmed as PHPT cases, with a total of 74 parathyroid lesions identified. As per patient‐based analysis, the diagnostic sensitivities of ^99m^Tc‐MIBI/^99m^TcO_4_^−^ dual‐isotope SPECT/CT fusion imaging and US were 94.12% (64/68) and 86.76% (59/68), respectively, with SPECT/CT demonstrating superior sensitivity. Per lesion‐based analysis revealed that SPECT/CT significantly outperformed US in both sensitivity (97.01% vs. 79.10%, *P *< 0.001) and PPV (85.87% vs. 80.72%, *P* = 0.006). Subgroup analysis showed that in group of the classical primary hyperparathyroidism (CPHPT), SPECT/CT exhibited higher sensitivity (96.22% vs. 84.91%, *P* < 0.001) and PPV (94.64% vs. 82.45%, *P* = 0.039) compared to US, whereas no significant difference was observed in group of the normocalcemic primary hyperparathyroidism (NPHPT). Additionally, in group of the CPHPT, moderate correlations were found between lesion volume and corrected serum calcium (Corr Ca) (*r* = 0.435, *P* = 0.003) as well as PTH levels (*r* = 0.303, *P* = 0.045).

**Conclusion:**

The dual‐isotope SPECT/CT fusion imaging has high diagnostic efficacy for PHPT, which can accurately locate lesions and provide reliable preoperative guidance for surgery. For patients with normal blood calcium but clinical symptoms of PHPT, combining this examination with ultrasound may offer complementary diagnostic value, although this finding requires validation in larger prospective studies. The degree of elevation in serum calcium and PTH levels can predict the volume and metabolic activity of parathyroid adenomas, and relevant biochemical indicators can guide the clinical screening of patients suitable for this examination.

## 1 BACKGROUND

Primary hyperparathyroidism (PHPT), one of the most prevalent endocrine disorders, is characterized by excessive secretion of parathyroid hormone (PTH) due to hyperfunctioning parathyroid glands.^1^ The clinical presentation typically includes elevated PTH levels, hypercalcemia, hypophosphatemia, and multi‐system involvement. With an estimated prevalence of 0.1%–0.7% in the general population,^2^ PHPT demonstrates significant epidemiological variations influenced by age, gender, and ethnic factors. Notably, postmenopausal women exhibit the highest disease susceptibility.^3^, ^4^ Currently recognized as the third most common endocrine disorder following diabetes mellitus and hyperthyroidism, PHPT represents a significant clinical entity in endocrine practice.

With the widespread adoption of serum PTH and calcium testing in clinical practice, a distinct PHPT variant termed normocalcemic primary hyperparathyroidism (NPHPT) has been increasingly recognized.^5^, ^6^ As a unique PHPT subtype differing from classical PHPT (CPHPT), NPHPT is characterized by elevated PTH levels with persistently normal serum calcium, presenting with or without typical clinical symptoms.^2^ However, the unclear pathophysiological mechanisms and indeterminate disease progression patterns have resulted in a lack of diagnostic consensus, frequently leading to delayed intervention and suboptimal clinical management for affected patients.^7^

Surgical intervention remains the primary treatment for PHPT, demonstrating significant therapeutic benefits including lesion eradication, symptom alleviation, and health restoration, with particular clinical value for NPHPT management.^8^ Current evidence highlights that surgical outcomes are substantially influenced by preoperative diagnostic accuracy, underscoring the critical importance of optimal imaging modality selection in clinical practice to enhance diagnostic precision prior to surgical planning.^9^

This retrospective study evaluated the diagnostic performance of ^99m^Tc‐MIBI/^99m^TcO_4_^−^ dual‐isotope SPECT/CT fusion imaging versus US for preoperative localization in suspected PHPT patients, while further investigating the correlation between parathyroid adenoma volume and serum biochemical markers to optimize clinical decision‐making.

## 2 METHODS

### 2.1 Patients

In total, 90 patients consecutively diagnosed with PHPT in our institution between September 2023 to October 2024. In accordance with the institutional review board, written informed consent was waived because of the retrospective study design.

**FIGURE 1:**
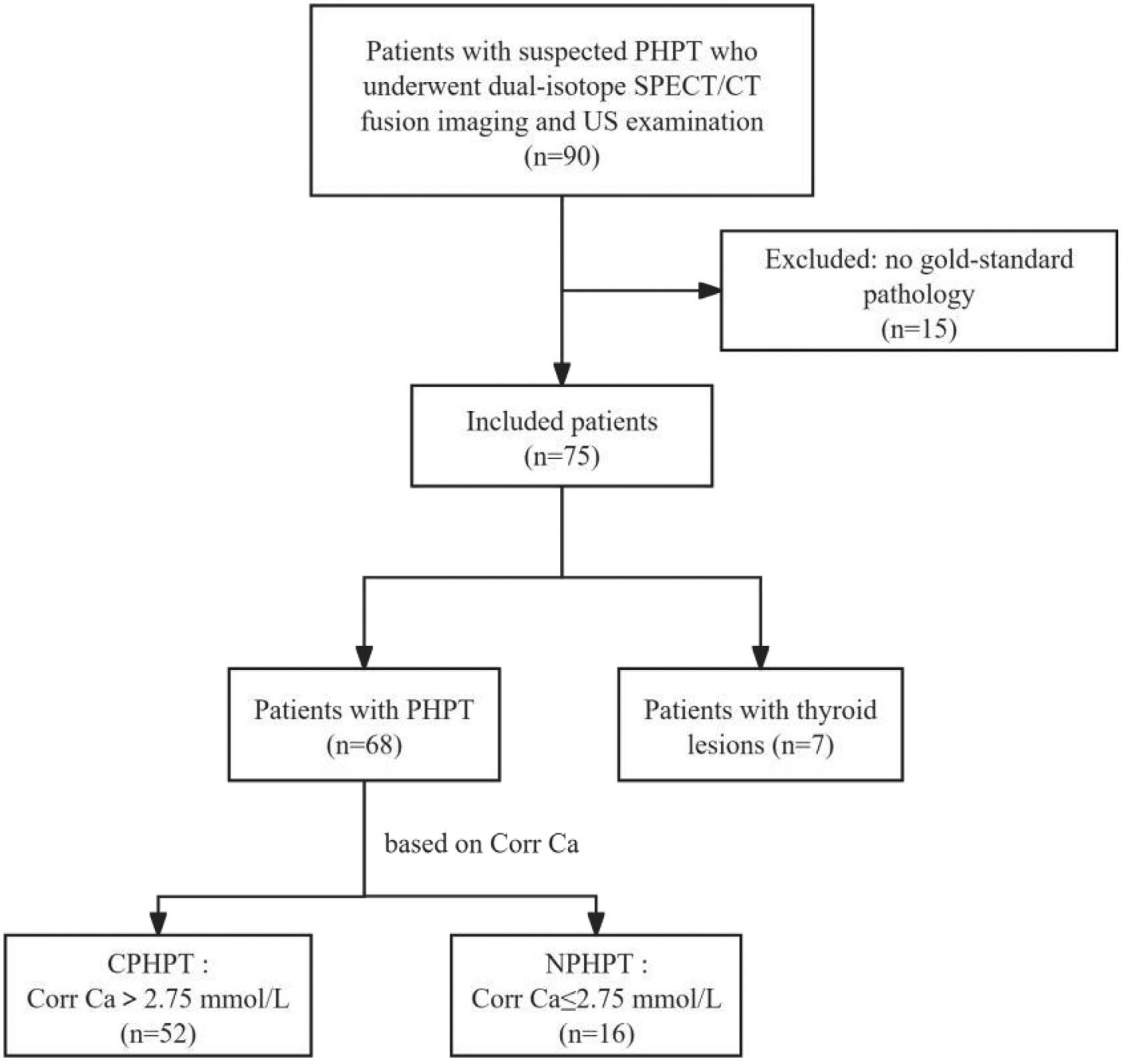
Flowchart of the study cohort.

Inclusion criteria were: (1) concurrent dual‐isotope SPECT/CT fusion imaging and US performed within a 2‐week interval; (2) undergoing parathyroid surgery with confirmed pathological results; (3) presence of significant clinical symptoms including lethargy, myalgia, dyspepsia, polyuria‐polydipsia, or muscle weakness, accompanied by elevated PTH levels regardless of calcium status. Exclusion criteria comprised: (1) previous neck surgery or malignancy under follow‐up; (2) secondary hyperparathyroidism due to renal disease; (3) parathyroidectomy performed more than 6 months after SPECT/CT examination; (4) incomplete or unavailable clinical data. Patient enrollment is illustrated in Figure 1.

### 2.2 US examinations

In our study, US were performed by one of three board‐certified radiologists with 5–15 years of experience in head and neck US. During US examination, patients were placed in the supine position with their neck exposed, and a Siemens Sequoia color Doppler US system equipped with a 10L4 linear array transducer (frequency range: 4.0–10.0 MHz) was used to systematically scan the neck region, meticulously documenting the echogenicity of thyroid tissue along with detailed characteristics of any abnormal echoes, including vascularity, echotexture, size, and morphological features.

A parathyroid lesion can be considered US positive if it appears as a homogeneous hypoechoic or isoechoic nodule in the expected migratory region of the parathyroid glands, demonstrating well‐defined margins, intact encapsulation, absence of surrounding tissue infiltration, and lack of corticomedullary differentiation.

### 2.3 Dual‐isotope SPECT/CT fusion imaging

**FIGURE 2:**
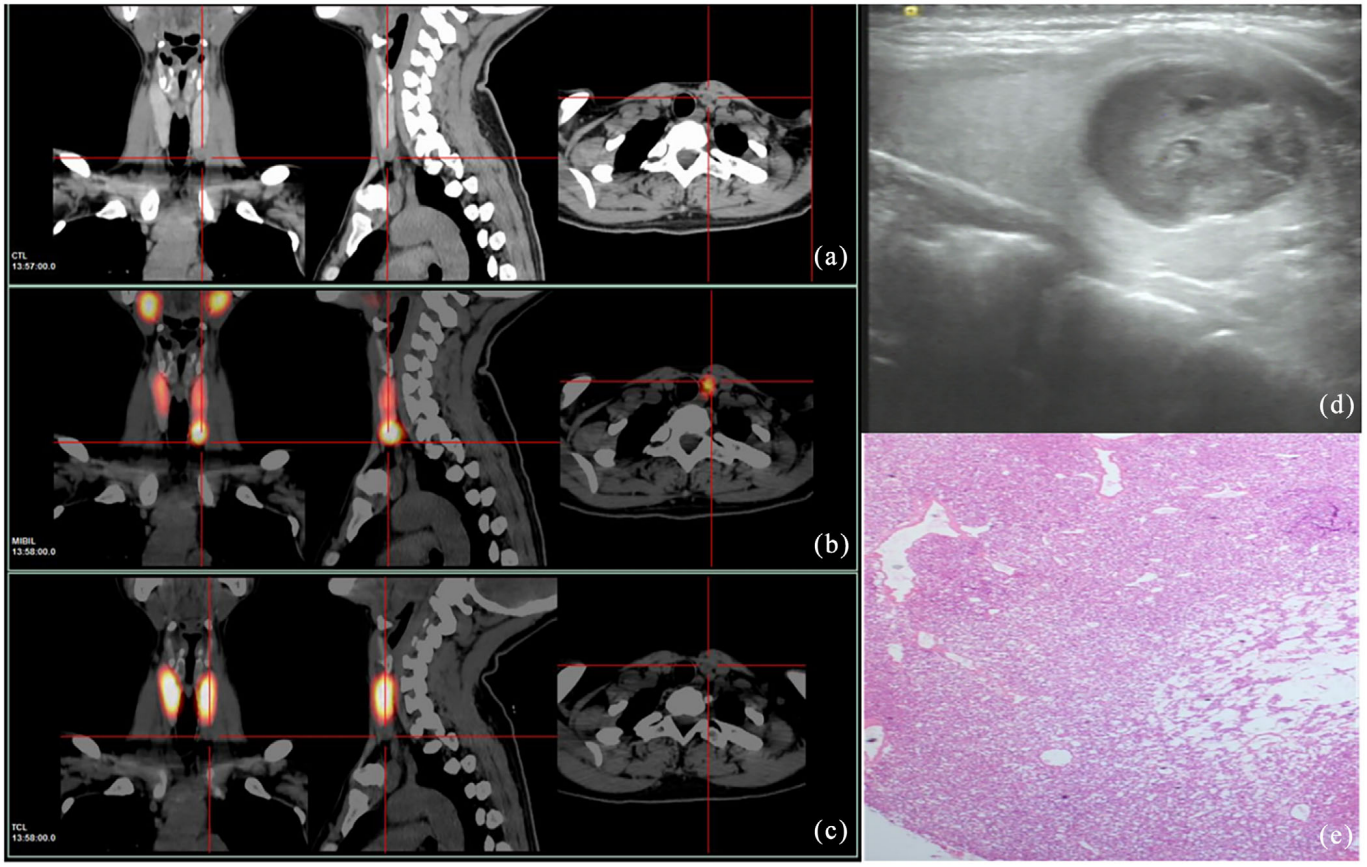
This patient suffered from dry mouth, dizziness, bone pain, and fatigue for more than 1 month due to unprovoked causes. Blood calcium: 2.71 mmol/L, PTH: 801.7pg/ml. Dual‐isotope SPECT/CT imaging: A round‐like soft tissue density lesion is visible on CT (a), with the lesion showing positive on ^99m^Tc‐MIBI images (b) and negative on ^99m^TcO_4_^−^ (c) images. In panels (a), (b), and (c), the lesion location is indicated by the red cross‐hairs. US suggests hypoechoic nodules behind the lower pole of the left lobe of the thyroid gland (d). The pathological results showed the parathyroid adenoma and surrounding thyroid tissue (e).

Dual‐isotope SPECT/CT fusion imaging was performed using a GE Discovery NM670 SPECT/CT system with ^99m^Tc‐MIBI and ^99m^TcO_4_^−^ (radiochemical purity > 95%, supplied by Beijing Atom High Tech Isotopes). Patients were positioned supine with neck fully exposed, and imaging covered the cervical region and upper mediastinum. After intravenous injection of 185 MBq (5 mCi) ^99m^TcO_4_^−^ for planar imaging (30 min acquisition), a second injection of 555–740 MBq (15–20 mCi) ^99m^Tc‐MIBI was administered 3–6 h later. Early‐phase (15 min) and delayed‐phase (120 min) planar images were acquired using a low‐energy high‐resolution parallel‐hole collimator (128 × 128 matrix, 140 keV photopeak, 20% window, 2× magnification, 150K counts per frame). Subsequent SPECT/CT fusion imaging employed 180° dual‐head rotation with 30 projection frames (15s per frame, 6° angular step), yielding a total of 60 SPECT projection views for reconstruction with CT parameters of 150 mA/120 kV (3 mm slice thickness, 512 × 512 matrix), and images were reconstructed using Xeleris processing software (Figure 2).

Two experienced nuclear medicine physicians independently analyzed the dual‐isotope SPECT/CT fusion images while remaining blinded to surgical outcomes, histopathological findings, US results, and serum calcium/PTH levels. Inter‐reader agreement was assessed using Cohen's kappa coefficient (*κ* = 0.87, indicating excellent agreement), and any discrepancies were resolved through consensus discussion, and the final reads were used for analysis. A lesion was considered positive if it met the following criteria: (1) a clearly defined soft‐tissue density nodule on CT, (2) definitive radiotracer uptake on ^99m^Tc‐MIBI imaging, and (3) absence of significant radiotracer uptake on ^99m^TcO_4_^−^ imaging.

### 2.4 Pathologic and biochemical analysis

The volume of resected parathyroid lesions was calculated using the ellipsoid formula: (a × b × c × π / 6) cm^3^, where a, b, and c represent the three‐dimensional measurements (in cm) of the gland.

Among the 68 patients confirmed with PHPT, subgroup stratification was performed based on corrected serum calcium (Corr Ca) levels to compare imaging performance between different phenotypes. The Corr Ca was calculated using the standard correction formula: Corr Ca (mmol/L) = measured serum Corr Ca (mmol/L) + [40 ‐ serum albumin (g/L)] × 0.02. Patients with Corr Ca > 2.75 mmol/L were classified as classical PHPT (CPHPT, *n* = 52), while those with values ≤2.75 mmol/L comprised the normocalcemic PHPT group (NPHPT, *n* = 16).

### 2.5 Statistical analysis

The statistical analyses were performed with the SPSS program version 26.0 for Windows. The results of the continuous variables are expressed as mean ± standard deviation and the qualitative variables as percentages. For statistical inference, chi‐square tests were employed. Using postoperative pathological results as the gold standard, we calculated the diagnostic sensitivity and PPV of two examination methods. Patient‐based paired diagnostic index comparisons were performed with McNemar's test, while clustered chi‐square test was adopted for lesion‐level analysis to eliminate interference from multiple lesions per single patient. Correlations between lesion volume and preoperative biochemical markers were assessed using Pearson correlation for normally distributed variables and Spearman correlation otherwise. The threshold of significance used for all the tests was *P* < 0.05.

## 3 RESULTS

This study enrolled 75 patients with suspected PHPT from our hospital, comprising 17 males and 58 females aged 17–76 years (mean age 52.26 ± 12.14 years). Among the 75 enrolled patients, postoperative pathological examination identified a total of 103 lesions. These included 74 parathyroid lesions, consisting of 50 parathyroid adenomas (60.98%), 15 atypical parathyroid adenomas (18.29%), 6 cases of parathyroid hyperplasia (7.32%), 2 parathyroid carcinomas (2.44%), and 1 parathyroid cyst (1.22%). The remaining 29 lesions were non‐parathyroid in origin, comprising 27 cases of simple nodular goiter (93.10%), 1 thyroid papillary carcinoma (3.45%), and 1 thyroid follicular tumor of uncertain significance (3.45%). Among the 68 patients with PHPT, 63 (92.65%) had a single lesion, while 5 (7.35%) exhibited multiple lesions. Additionally, 19 patients had concurrent thyroid disease. This discrepancy arises because some patients had multiple lesions. Table 1 summarizes the baseline characteristics, median serological markers, and anatomical locations of the parathyroid lesions.

**TABLE 1:** Patient characteristics.

Parameter	Value
Age (years): median (IQR)	51.7 (IQR: 47–60)
Gender *N* (%)	
Female	58 (77.3%)
Male	17 (22.7%)
Preoperative serum tests: median (IQR)	
Ca (mmol/L)	2.74 (IQR: 2.64–3.08)
Cl (mmol/L)	107.30 (IQR: 105.70–108.00)
P (mmol/L)	0.92 (IQR: 0.77–0.96)
PTH (pg/mL)	367.19 (IQR: 145.3–373.9)
Surgical localization, No. (%)	
Left inferior	21 (28.4%)
Left superior	8 (10.8%)
Right inferior	39 (52.7%)
Right superior	6 (8.1%)

Preoperative laboratory parameters included serum calcium levels ranging from 2.21 to 3.95 mmol/L (mean 2.83 ± 0.38 mmol/L), serum chloride levels ranging from 98.00 to 125.10 mmol/L (mean 107.06 ± 3.71 mmol/L), serum phosphorus levels ranging from 0.37 to 1.95 mmol/L (mean 0.87 ± 0.25 mmol/L), and PTH levels ranging from 72.29 to 3808.00 pg/ml (mean 419.67 ± 604.20 pg/ml). The volume of parathyroid lesions varied between 0.07 to 26.71 cm^3^ (mean 2.77 ± 4.67 cm^3^).

### 3.1 Dual‐isotope SPECT/CT fusion imaging vs. US for PHPT lesion detection

Patient‐based analysis showed diagnostic sensitivities of 94.12% (64/68) for dual‐isotope SPECT/CT fusion imaging and 86.76% (59/68) for US in PHPT patients, with no statistically significant difference (χ^2^ = 2.78, *P *= 0.096); however, the limited sample size at the patient level may have reduced statistical power, and lesion‐based analysis better reflects the localization capability of the two modalities. Lesion‐based analysis demonstrated superior localization by dual‐isotope SPECT/CT fusion imaging (66 lesions) versus US (55 lesions). As shown in Table 2, dual‐isotope SPECT/CT fusion imaging demonstrated significantly higher sensitivity (97.01% vs. 79.10%, *P* < 0.001) and PPV (85.87% vs. 80.72%, *P* = 0.006) compared to US. Figure 2 illustrates a representative true‐positive case.

**TABLE 2:** Sensitivity and PPV of dual‐isotope SPECT/CT fusion imaging and US.

		US	SPECT/CT	P
All patients	Sensitivity	79.10%	97.01%	<0.001^*^
	PPV	80.72%	85.87%	0.006^*^
NPHPT	Sensitivity	68.75%	93.75%	0.295
	PPV	68.42%	84.21%	0.370
CPHPT	Sensitivity	84.91%	96.22%	<0.001^*^
	PPV	82.45%	94.64%	0.039^*^
All patients (Combined)	Sensitivity	98.06% 89.00%		——
	PPV			——

Note: All data are lesion‐level analyses. NPHPT subgroup: 16 patients (all with single lesion, 16 lesions in total); CPHPT subgroup: 52 patients (66 lesions in total).

^*^Significant with *P*<0.05.

### 3.2 Dual‐isotope SPECT/CT fusion imaging vs. US in detecting lesions in CPHPT and NPHPT

In group of the CPHPT, dual‐isotope SPECT/CT fusion imaging demonstrated significantly higher sensitivity (96.22% vs. 84.91%, *P* < 0.001) and PPV (94.64% vs. 82.45%, *P* = 0.039) than US. For the group of the NPHPT, dual‐isotope SPECT/CT fusion imaging showed numerically superior but non‐significant improvements in sensitivity (93.75% vs. 68.75%, *P* = 0.295) and PPV (84.21% vs. 68.42%, *P* = 0.370). Combined modality achieved 94.74% sensitivity (Table 2).

For lesion‐based analysis, the combined modality (positive by either SPECT/CT or US) achieved a sensitivity of 98.60% (73/74) and a PPV of 89.0.% (73/82).

### 3.3 Correlation between preoperative serum markers and resected lesion volume

**FIGURE 3:**
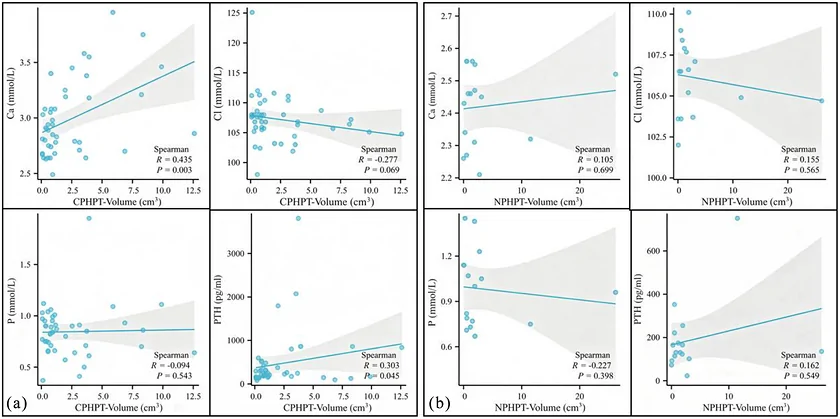
Correlation between parathyroid lesion volume(cm^3^) and serum Corr Ca(mmol/L) /P(mmol/L) /Cl(mmol/L) /PTH(pg/ml) levels in CPHPT (A) and NPHPT (B) groups. *Note: The blue line represents the linear regression fit, and the gray shaded area indicates the 95% confidence interval of the regression.

In CPHPT patients, lesion volume showed moderate correlations with serum Corr Ca (*r* = 0.435, *P* = 0.003) and PTH (*r* = 0.303, *P* = 0.045), but not with chloride or phosphorus (Figure 3a). No significant correlations were observed in NPHPT patients (Figure 3b, TABLE 3).

**TABLE 3:** The correlation between parathyroid lesion volume and blood biochemical indicators.

Group		Ca	Cl	P	PTH
NPHPT	r	0.105	0.155	−0.227	0.162
	*P*	0.699	0.565	0.398	0.549
CPHPT	r	0.435	−0.277	−0.094	0.303
	*P*	0.003^*^	0.069	0.543	0.045^*^

^*^Significant with *P* < 0.05.

## 4 DISCUSSION

This study demonstrates the superior diagnostic value of ^99m^Tc‐MIBI/^99m^TcO_4_^−^ dual‐isotope SPECT/CT for preoperative localization in PHPT, particularly showing significantly higher sensitivity than US in CPHPT cases. Notably, positive correlations between lesion volume and serum Corr Ca/PTH levels in CPHPT patients may guide optimal preoperative planning and surgical exploration.

To our knowledge, this study offers several unique contributions to the existing literature from a medical physics perspective. First, while prior studies have focused on CPHPT, limited data exist on the diagnostic performance of dual‐isotope SPECT/CT specifically in the NPHPT subgroup; our findings help fill this gap. Second, we provide a detailed analysis of false‐positive findings, offering insights into potential diagnostic pitfalls. Third, from a medical physics perspective, we contextualize the known resolution limits of SPECT/CT in detecting sub‐centimeter parathyroid lesions, providing a practical benchmark for interpreting small‐lesion detection based on established physical principles.

Dual‐isotope SPECT/CT fusion imaging is a diagnostic tool that integrates functional and anatomical imaging, enabling the visualization of lesions along with their spatial relationships to adjacent tissues such as the thyroid, thereby providing precise localization of pathological foci. The dual‐isotope SPECT/CT fusion imaging using ^201^TI/^99m^TcO_4_^−^ demonstrates high sensitivity in diagnosing PHPT. However, due to the limitations of ^201^TI including its cyclotron‐dependent production, high cost, low gamma energy (which compromises detection efficiency), and relatively high radiation dose. Geatti et al. pioneered the alternative use of ^99m^Tc‐MIBI/^99m^TcO_4_^−^ dual‐isotope SPECT/CT fusion imaging. Their findings revealed significantly improved diagnostic sensitivity with ^99m^Tc‐MIBI compared to ^201^TI.^10^, ^11^, ^12^ Nuclear imaging holds substantial diagnostic value for PHPT as a functional modality, wherein only hyperfunctioning parathyroid glands are visualized, ensuring objective results. Normal parathyroid glands, owing to their small size, low blood flow, and minimal cellular activity, typically remain undetectable. Imaging becomes feasible only when parathyroid hyperactivity occurs.^13^, ^14^

Dual‑isotope SPECT/CT imaging with ^99m^Tc‑based tracers presents several technical challenges. First, since both ^99m^Tc‑MIBI and ^99m^TcO_4_^−^ emit gamma rays at the identical photopeak energy of 140 keV, classic inter‑nuclide spectral cross‑talk is avoided when both tracers are ^99m^Tc‑labeled; however, careful energy‑window calibration and scatter correction are still required to minimize Compton scatter contamination and ensure accurate thyroid background subtraction. Second, the subtraction algorithm assumes stable thyroid uptake of ^99m^TcO_4_^−^ between early and delayed phases; this may be compromised in patients with thyroid nodules or hyperfunctioning tissue, potentially resulting in incomplete background removal.^15^ Third, the SPECT system, equipped with a low‑energy high‑resolution (LEHR) collimator and measured at a typical parathyroid imaging distance (10 cm from the collimator surface), has a system spatial resolution of approximately 8–10 mm FWHM—determined by the collimator rather than the gamma camera's intrinsic resolution (around 3 mm for ^99m^Tc).^16^, ^17^, ^18^ According to the partial volume effect principle, when lesion size is < 1.5–2× the system FWHM (< 12–16 mm), tracer uptake is substantially diluted by adjacent tissue activity; lesions < 5.0 mm are generally undetectable on SPECT.^19^, ^20^ To partially mitigate these limitations, delayed‑phase imaging was acquired at 120 min post‑injection to maximize the target‑to‑background ratio, and iterative reconstruction with resolution recovery was applied (OSEM, 2 iterations, 10 subsets).

US serves as a routine screening and diagnostic tool for parathyroid disorders, offering notable advantages in detecting parathyroid adenomas, with a reported sensitivity ranging from 50% to 93%.^19^, ^21^ Its detection rate primarily depends on gland size and the density contrast with surrounding tissues, though results are highly operator‐dependent. Post‐thyroidectomy challenges such as surgical scarring, fibrosis, and local edema—further compromise US resolution by obscuring normal anatomical landmarks (e.g., the thyroid capsule and tracheoesophageal groove), thereby complicating the identification of PHPT lesions.

In this study, we evaluated the preoperative localization efficacy of US versus dual‐isotope SPECT/CT fusion imaging in PHPT patients. Our findings demonstrate that fusion imaging surpasses US in diagnostic accuracy, aligning with Giacomo Pata's report of a 79%–89% positive predictive value for dual‐isotope SPECT/CT fusion imaging in PHPT detection.^22^

Lesion size is a primary determinant affecting detection sensitivity in both US and dual‐isotope SPECT/CT fusion imaging.^23^ In our study, US failed to detect 7 PHPT lesions with volumes < 1.0 cm^3^, while dual‐isotope SPECT/CT fusion imaging demonstrated superior diagnostic capability for smaller lesions, missing only one case with diameter < 5.0 mm. This improved performance notwithstanding, dual‐isotope SPECT/CT fusion imaging still faces limitations due to the relatively low spatial resolution of SPECT, resulting in reduced sensitivity for minute lesions particularly when lesions are adjacent to high‐uptake regions near the thyroid.^24^ As noted above, the physical limitations of SPECT system resolution (8–10 mm FWHM) inherently constrain the detection of lesions < 5.0 mm, explaining the single false‐negative case in our cohort. The partial volume effect significantly impacts small lesion detection: when adenoma size approaches the resolution limits of dual‐isotope SPECT/CT fusion imaging, tracer uptake may be diluted by surrounding tissue signals, potentially causing false‐negative results. Furthermore, even in metabolically active lesions (such as those with vigorous PTH secretion), insufficient tracer accumulation or rapid washout can lead to low total counts, further contributing to false‐negative findings. These technical limitations underscore the challenges in detecting subcentimeter parathyroid lesions, despite the overall advantage of dual‐isotope SPECT/CT fusion imaging over US. Contrary to the findings by Neuberger et al. which showed reduced PTH levels in scan‐negative patients, our data did not reveal this inverse relationship.^25^

On the other hand, previous studies have indicated that the presence of thyroid diseases can impair the ability of SPECT scans to localize lesions preoperatively, while US demonstrates distinct advantages. The dual‐isotope subtraction technique relies on the linear combination of early and delayed images to remove thyroid background activity. However, this approach assumes that no significant washout of ^99m^Tc‐MIBI occurs from thyroid tissue. In patients with thyroid nodules, especially those containing oxyphilic cells rich in mitochondria, delayed washout of ^99m^Tc‐MIBI violates this assumption, leading to incomplete subtraction and false‐positive readings. This represents a fundamental physical limitation of the dual‐isotope subtraction method that cannot be fully overcome by algorithmic improvements alone.

**FIGURE 4:**
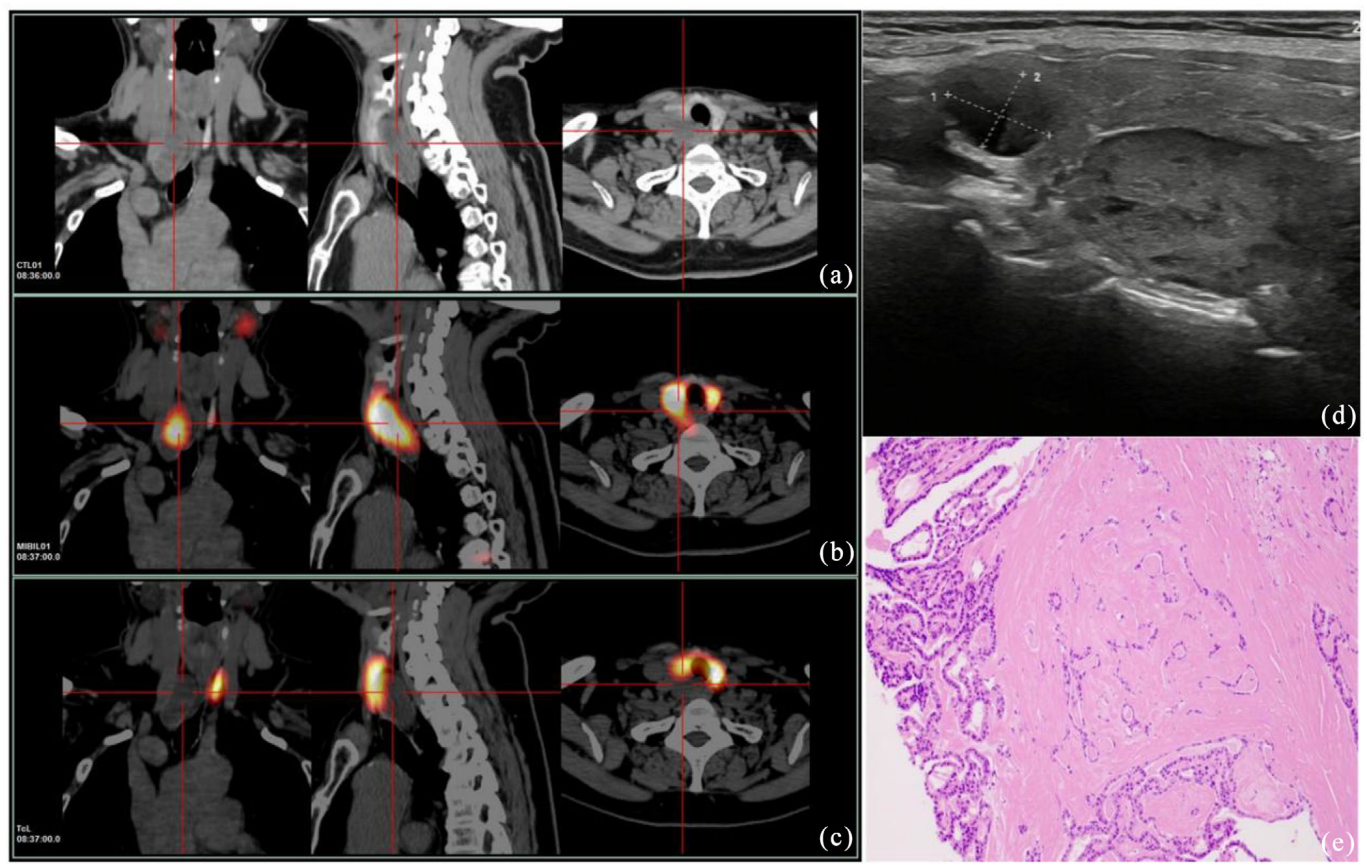
A patient has been in the hospital for nearly a month due to fatigue, dizziness, and poor appetite. Dual‐isotope SPECT/CT imaging: A round‐like soft tissue density lesion is visible on CT (A), with the lesion showing positive on ^99m^Tc‐MIBI images (B) and negative on ^99m^TcO_4_^−^ (C) images. In panels (A), (B), and (C), the lesion location is indicated by the red cross‐hairs. US shows nodules in the right lobe of the thyroid gland, possibly parathyroid adenoma (D). The final postoperative pathology confirmed that it was an undeterminable follicular tumor of the thyroid gland (E).

In our study, thyroid disorders such as thyroid adenoma, nodular goiter, and thyroid carcinoma comprising oxygenophilic cells rich in mitochondria also exhibit uptake of ^99m^Tc‐MIBI. Due to the delayed washout of ^99m^Tc‐MIBI in these conditions, the imaging signals cannot be effectively subtracted, potentially leading to false identification as parathyroid nodules and consequently reducing diagnostic sensitivity (Figure 4).^26^, ^27^ This interference may compromise the accuracy of dual‐isotope SPECT/CT fusion imaging (Figure 4). Postoperative pathology in 19 patients revealed PHPT coexisting with thyroid nodules, 16 of whom presented with hypercalcemia. The mechanism underlying PHPT concurrent with thyroid diseases remains unclear but may involve PTH stimulated synthesis of insulin like growth factor‐1, promoting thyroid cell proliferation.^28^ Alternatively, some researchers hypothesize that thyroid lesions increase calcitonin secretion from C‐cells, antagonizing PTH and subsequently triggering compensatory hyperfunction of the parathyroid glands.^29^ US offers significant advantages in evaluating thyroid lesions. Combining this modality with dual‐isotope SPECT/CT fusion imaging can improve differentiation between thyroid lesions and parathyroid lesions coexisting with thyroid abnormalities, thereby enabling a more comprehensive assessment of PHPT patients.^28^, ^30^

NPHPT, a distinct subtype of PHPT, has gained increasing clinical recognition in recent years, with surgical intervention remaining the primary treatment approach.^31^, ^32^ While numerous imaging studies have investigated preoperative localization for CPHPT patients, research focusing specifically on NPHPT cases remains limited. In our study, patients were stratified into two groups based on serum Corr Ca levels. The results demonstrated that dual‐isotope SPECT/CT fusion imaging showed statistically superior sensitivity and PPV over US in CPHPT patients; although numerically higher indexes were found in the NPHPT group, no statistical difference existed between the two imaging modalities (both *P* > 0.05). The lack of significance is likely attributable to the limited sample size of 16 patients in this subgroup. Despite the lack of statistical significance, the numerically superior performance of SPECT/CT in the NPHPT subgroup suggests a potential benefit. However, given the small sample size, this should be considered a hypothesis‐generating finding that warrants further investigation in larger, adequately powered studies.^33^ These findings suggest that for patients with elevated PTH but normal serum Corr Ca levels, routine preoperative evaluation combining dual‐isotope SPECT/CT fusion imaging with US can significantly improve parathyroid disease characterization and optimize surgical planning.

PTH level serves as the primary basis for diagnosing PHPT and guiding treatment decisions, while also representing a crucial indicator of disease severity. Previous studies have demonstrated a positive correlation between SPECT imaging positivity rates and both serum PTH levels and lesion volume, with elevated Corr Ca levels further reflecting disease severity.^34^ Although Neuberger et al. reported that imaging‐negative patients typically exhibit lower PTH levels, our study failed to replicate this association.^35^

Our analysis revealed significant correlations between serum PTH/Corr Ca levels and total gland volume in the CPHPT cohort. These correlations are modest and exploratory. They suggest an association between PTH/Corr Ca elevation and pathological gland volume, a hypothesis that requires validation in larger prospective studies to confirm clinical utility.^32^, ^36^, ^37^ The observed relationship between preoperative PTH/Corr Ca levels and parathyroid adenoma volume carries particular clinical relevance for cases where biochemical markers suggest a large adenoma despite small gland size. In such scenarios, additional preoperative or intraoperative localization studies become essential to exclude larger occult lesions.

These findings further support the hypothesis that NPHPT and CPHPT are distinct disease entities with different underlying mechanisms. Specifically, NPHPT might not be mediated through calcium homeostasis but rather through phosphate‐regulated PTH activity, alternative PTH secretory pathways, or yet unidentified mechanisms.

The moderate correlation between lesion volume and serum Corr Ca/PTH levels in CPHPT patients may reflect that more metabolically active parathyroid tissue (producing higher hormone levels) is associated with larger gland size. In NPHPT patients, the lack of correlation may be due to milder metabolic activity or intermittent hormone secretion. Regarding cost, while dual‐isotope SPECT/CT is more expensive than US, the improved diagnostic accuracy may reduce failed surgeries and reoperations, potentially offsetting the initial higher cost.

Beyond clinical comparison, this study provides several practical insights for medical physicists optimizing parathyroid SPECT/CT protocols. First, we explicitly quantify the impact of SPECT system spatial resolution (8–10 mm FWHM) and the partial volume effect on the lower limit of parathyroid lesion detectability (almost 5 mm), offering a physical benchmark for interpreting false‐negative findings in small lesions. Second, we highlight a fundamental limitation of the dual‐isotope subtraction technique: the assumption of stable thyroid background activity is violated in patients with mitochondrial‐rich thyroid nodules, leading to incomplete signal subtraction. This physical constraint is independent of post‐processing algorithms and is crucial for medical physicists to understand when optimizing protocols. Third, our correlation analysis suggests that preoperative PTH and calcium levels, while not replacing imaging, can provide a probabilistic guide to lesion size, potentially informing the selection of imaging sequences or reconstruction parameters. These findings directly support medical physicists in protocol optimization, image interpretation, and interdisciplinary communication regarding the technical capabilities and limitations of parathyroid SPECT/CT.

However, our study has several limitations: (1) it only included patients with PHPT, excluding those with secondary or tertiary PHPT; (2) the sample size was relatively small; (3) as a single‐center retrospective study, potential selection bias may exist; (4) the CPHPT subgroup(defined as Corr Ca > 2.75 mmol/L) included 52 patients, while the NPHPT subgroup included 16 patients; the small sample size of the NPHPT subgroup limits the statistical power of subgroup comparisons and may have contributed to the lack of significant differences in some analyses; and (5) because only patients who underwent surgery (and thus had pathological confirmation) were included, our cohort is enriched with imaging‐positive cases. Consequently, our reported sensitivity and PPV may be overestimated, and the findings may not be generalizable to patients managed non‐operatively.

## 5 CONCLUSION

In conclusion, dual‐isotope SPECT/CT fusion imaging demonstrates significantly higher sensitivity and diagnostic accuracy than US in detecting CPHPT. For the smaller NPHPT subgroup, while SPECT/CT showed numerically superior performance, the difference was not statistically significant, likely due to limited sample size. Therefore, no statistically significant diagnostic advantage of SPECT/CT was verified in NPHPT due to the limited sample size; future large‐scale prospective studies are required to confirm its potential incremental diagnostic value. The combined use of dual‐isotope SPECT/CT and US further enhances comprehensive evaluation, particularly in patients with concurrent thyroid diseases.

## AUTHOR CONTRIBUTIONS

YL and HG contributed to the study concept and design. ND and ZZ retrieved and filtered the articles. YL and XL extracted the data. YL and YL analyzed the data. YL, ZL and PF interpreted the data. YL, ND drafted the manuscript. YL, PF and HG contributed to the critical revision of the manuscript. All authors contributed to the paper and approved the submitted version.

## CONFLICT OF INTEREST STATEMENT

The authors declare no conflicts of interest.

## Data Availability

Access to unpublished data from our study is subject to negotiation with The First Affiliated Hospital of Harbin Medical University, or as may be required by applicable law.
